# Molecular detection tool for prompt, reliable and precise diagnosis of the rice weevil, *Sitophilus oryzae* (Linnaeus) infestation in wheat

**DOI:** 10.3389/fpls.2025.1628692

**Published:** 2025-08-18

**Authors:** Kanika Nagpal, Poonam Jasrotia, Maha Singh Jaglan, Prem Lal Kashyap, Sunny Maanju, Sudheer Kumar

**Affiliations:** ^1^ Crop Protection Division, Indian Council of Agricultural Research (ICAR)- Indian Institute of Wheat and Barley Research, Karnal, Haryana, India; ^2^ Department of Entomology, College of Agriculture, Chaudhary Charan Singh (CCS) Haryana Agricultural University, Hisar, Haryana, India

**Keywords:** cereals, post-harvest losses, stored grain pests, pest detection, molecular diagnostics

## Abstract

The rice weevil (*Sitophilus oryzae* L.) is one of the most destructive pests of stored cereal grains, particularly wheat, leading to considerable post-harvest losses and posing serious threats to global food security and international trade. Rapid and accurate identification of infestations is essential for implementing timely pest management strategies and adhering to phytosanitary regulations. In this study, we report the development and validation of a molecular diagnostic assay that is rapid, sensitive, and highly specific for the early detection of *S. oryzae* in stored wheat grains. Two novel species-specific oligonucleotide primer sets—KNSoCox1F1/KNSoCox1R1 and KNSoCox2F1/KNSoCox2R1—were designed to amplify target regions of the *mitochondrial cytochrome oxidase subunits I and II* (*COI* and *COII*), generating diagnostic fragments of 176 bp and 248 bp, respectively. Conventional PCR demonstrated high specificity, with no cross-reactivity observed in other non-target insects or uninfested wheat samples. Further, sensitivity assessments using quantitative real-time PCR (qPCR) revealed detection thresholds as low as 1 picogram of genomic DNA, which corresponds to a single insect per 10 kg of grains. The assay easily operates in moderately equipped molecular laboratories and offers quick results with streamlined workflows or automation, making it ideally suited for use in quarantine stations, grain storage facilities, and entomological diagnostic laboratories. Its reliability, speed, and cost-efficiency make it a powerful tool for pest surveillance, ecological studies, and enhancing biosecurity protocols.

## Introduction

Wheat (*Triticum aestivum* L.) is a globally significant staple crop, cultivated extensively to meet the escalating food demands driven by the rapid growth of the human population (Nikos and Jelle, 2012; Shiferaw et al., 2013). Due to its widespread cultivation and substantial nutritional contribution, wheat is a cornerstone of global food and nutritional security and is regarded as the most strategically vital cereal crop worldwide (Nyaupane et al., 2024; Ulukan, 2024). It serves as a principal dietary component for approximately 40% of the global population. With the world population projected to reach 9.8 billion by 2050 (Severo et al., 2024), agricultural systems must either augment crop production by an estimated 1 billion tons annually (FAO, 2015) or substantially minimize post-harvest losses (Iqbal et al., 2017) to ensure sustainable food availability. Reduction of post-harvest losses could significantly enhance global food reserves, thereby diminishing the need for further intensification of agricultural practices (Kiaya, 2014).

Stored grain pests are responsible for approximately 20–40% of global post-harvest grain losses (Igbeka, 2013), posing a serious challenge to food availability and nutritional security (Midega et al., 2016). Among the key pests of stored cereals, the rice weevil, *S. oryzae* (L.) (Coleoptera: Curculionidae) is particularly destructive. Although traditionally associated with rice, *S. oryzae* is a polyphagous species capable of infesting wheat and other cereal grains. The insect completes its development within the seed kernels, resulting in concealed infestations that can lead to significant economic losses and pose health risks such as allergic reactions and gastrointestinal disturbances (Hansen et al., 2012).

Timely and accurate detection of *S. oryzae* infestations is essential for implementing effective management strategies (Obrepalska-Steplowska et al., 2008). Traditional detection methods have relied on sensory evaluation (e.g., visual inspection, odor assessment), temperature monitoring in storage units (Abass et al., 2018), and various physical and chemical techniques, including kernel staining (Frankenfeld, 1948), near-infrared spectroscopy (Dowell et al., 1998), acoustic sensors (Hagstrum et al., 1996; Fleurat-Lessard et al., 2006), microwave radar (Mankin, 2004), X-ray imaging (Karunakaran et al., 2003; Fornal et al., 2007), immunoassays (Kitto et al., 1994), pitfall traps, and visual surveillance (Njoroge et al., 2019). Despite their utility, many of these methods are labor-intensive, costly, time-consuming, and limited by low sensitivity, particularly in detecting juvenile insect stages or differentiating morphologically similar species. Acoustic techniques, for instance, necessitate specialized equipment for capturing insect-generated sounds (Mankin et al., 2021). Additionally, environmental sensing and volatile compound analysis using electronic-nose (e-nose) technology (Kaushik and Singhai, 2018) have been explored; however, these methods are often compromised by external environmental variables and remain inadequate for detecting cryptic or early-stage infestations (Neethirajan et al., 2007).

In this regard, molecular diagnostics offer a promising alternative for the rapid and precise identification of stored grain pests. These techniques can detect both active infestations and residual genetic material from past infestations (Křížková-Kudlíková and Hubert, 2007). Given the growing emphasis on automation and time-efficient quality control in food processing industries, molecular tools, particularly those based on Polymerase Chain Reaction (PCR), offer enhanced sensitivity and specificity. They facilitate early detection across all life stages of insects and can also elucidate genetic relationships, species distribution, and intra-species diversity (Fleurat-Lessard and Pronier, 2006).

Among molecular methods, PCR—especially quantitative real-time PCR (qPCR)—has gained attraction. While PCR is routinely employed in the food industry for detecting pathogens and genetically modified organisms (GMOs), its application for insect detectionin grains is gaining broader acceptance now-a-days in pest monitoring and quarantine practices. Several studies have successfully employed PCR to detect and differentiate stored grain insect pests. For example, species-specific DNA markers have been used to detect early-stage infestations of *Rhyzopertha dominica* in wheat (Negi et al., 2021), while multiplex PCR has facilitated differentiation among *S. oryzae*, *S. granarius*, and *S. zeamais* (Solà et al., 2018). Amplification of *mitochondrial cytochrome oxidase I* (*COI*) gene regions has proven effective for identifying species such as *Sitophilus* spp., *R. dominica*, *Plodia interpunctella*, *Oryzaephilus* spp., and *Samea* spp (Abbasi et al., 2021). Specific primers targeting mitochondrial genes like *mtCOI* and *mtCOII*, as well as internal transcribed spacer (ITS) regions of rDNA, have been developed for both standard and real-time PCR applications (Obrepalska-Steplowska et al., 2008; Nowaczyk et al., 2009). These tools allow accurate and stage-independent detection of pest species, including *S. oryzae* and *S. zeamais* (Devi et al., 2017). Recent work by Negi et al. (2021) demonstrated the application of qRT-PCR targeting *mtCOI* for rapid and high-fidelity detection of *Tribolium castaneum*, highlighting its potential for use in grain trade, milling, baking, and food processing industries.

Molecular techniques are now recognized for their high accuracy, specificity, sensitivity, and throughput capacity. A critical advantage lies in their ability to detect both active and historical infestations, enabling timely and informed management decisions. PCR and RT-PCR, in particular, are poised to play a pivotal role in the early detection of *S. oryzae*, thereby mitigating post-harvest losses. In light of the existing knowledge gaps and practical constraints of conventional methods, the present study was designed to develop species-specific primers targeting mitochondrial cytochrome oxidase regions, with the objective of establishing a simple, sensitive, and rapid molecular diagnostic protocol for *S. oryzae* detection in wheat grains.

## Materials and methods

### Wheat (*T. aestivum* L.) germplasm collection

The experiments were conducted during 2021–22 in the Entomology Laboratory of the Crop Protection Division at ICAR–Indian Institute of Wheat and Barley Research (IIWBR), Karnal, India. Disease- and insect-free wheat germplasm of the cultivar HS490, essential for the study, was obtained from the Germplasm Resources Unit (GRU) at ICAR–IIWBR. Prior to experimentation, the grains were thoroughly cleaned and inspected to eliminate any damaged kernels, thereby ensuring the absence of contamination.

### Maintaining *S. oryzae* pure culture

The initial stock population of *S. oryzae* adults used in the study was sourced from the experimental laboratory of ICAR–IIWBR, Karnal, and subsequently reared for several generations on healthy wheat grains in the Insect Rearing Room of the Entomology Laboratory. For experimental purposes, the *S. oryzae* culture was maintained on undamaged wheat grains stored in 1-liter glass jars (20 cm height x 5 cm diameter) under controlled environmental conditions within a Biochemical Oxygen Demand (BOD) incubator, set at a temperature of 28 ± 2°C and relative humidity of 70 ± 5%.

### Genomic DNA isolation from *S. oryzae* and wheat grains

Genomic DNA was extracted from adult *S. oryzae* individuals (used as positive controls) obtained from the maintained pure culture. Insect tissues were lysed using TNES buffer (comprising Tris-HCl, NaCl, EDTA, and SDS) in a microtube homogenizer. The resulting DNA pellet was resuspended in 1% TE (Tris-EDTA) buffer. To eliminate RNA contamination, 3 µl of RNase solution (10 mg/ml) was added to each sample, followed by incubation in a water bath at 37°C for 30 minutes. Additionally, genomic DNA was isolated by crushing the whole grains (5 g) of the wheat genotype HS490 and then following modified CTAB (cetyltrimethylammonium bromide) extraction protocol, based on the method described by Saghai-Maroof et al. (1984), with slight modifications.

Furthermore, genomic DNA was also isolated from contaminated wheat grains. For contaminating the wheat grains, two hundred gram of wheat grains, in triplicate, after conditioning, were maintained at 25 ± 2°C and 65 ± 5% relative humidity in round plastic containers of capacity 500 grams. One hundred pair of *S. oryzae* adults, were transferred in each container holding the grains and was covered with muslin cloth and tightened using rubber bands. Contaminated and infested grains were sub-sampled (5 g) for DNA isolation at 30 days after infestation through the same modified CTAB extraction method, resulting in contamination level of 1000 insects in one kg of grains.

### DNA quantity and quality

The quantity and purity of DNA was tested using BioDrop Touch PC + Spectrophotometer (BioDrop, Cambridge shire, UK) by loading 1 μl sample of stock DNA. It was later diluted to prepare the working solution of 50 ng/μl concentration using the NFW (Nuclease Free Water) for further PCR based assays.

### DNA amplification

For molecular identification of *S. oryzae*, genomic DNA of the insects was amplified *in vitro* in thermocyclers using already reported and available specific primers from the literature. Initially, four different primers (SOF1-SOR1, SOF2-SOR2, SOF3-SOR3, and SOF4-SOR4) were assessed for *S. oryzae*, that amplified the cytochrome oxidase (*COX*) gene of *S. oryzae* (**Table 1**). These primers were then used to amplify *COI* gene from extracted DNA of adult insects isolated from infested wheat grain samples.

**Table 1:** Brief description of already reported *S. oryzae* specific primers used in the present investigation.

Sr. no.	Primer name	Forward and reverse primer sequences (5’-3’)	Amplicon size (bp-base pairs)	T_a_ -Annealing temperature (°C)
1.	SOF1	AGTTTGCTAATTCGGGCAGA	950	55.5
	SOR1	ACTCCGGTTAATCCTCCAAT		
2.	SOF2	CTAATTCGGGCAGAACTAGGAA	484	55.5
	SOR2	AGAGGAGGAGAATAGCAGTGATTCTT		
3.	SOF3	TTTCTTCAAGATAGAGCCTCACC	551	56.5
	SOR3	GCTCCGCAAATTTCAGAACA		
4.	SOF4	CTACTAACCACAAAGATATCGG	653	50.0
	SOR4	TAAACTTCAGGGTGACCAAAAAATCA		

The PCR amplification was performed in a total reaction volume of 10 µL, comprising 1 µL of template DNA, 1 µL of primer mix (containing equimolar concentrations of both forward and reverse primers, each at 10µM concentration), 5 µL of GoTaq^®^ G2 Green Master Mix (Promega), and 3 µL of nuclease-free water (NFW). The thermocycling conditions included an initial denaturation step at 95°C for 5 minutes, followed by 35 cycles of denaturation at 94°C for 30 seconds, annealing at the primer-specific annealing temperature (Ta) for 30 seconds, and extension at 72°C for 1 minute. A final extension was carried out at 72°C for 10 minutes, and the amplified products were subsequently held at 4°C. Reactions were conducted using a Q Cycler 96 thermal cycler (Hain Life Sciences, UK).

The resulting PCR products were resolved by electrophoresis on a 2% agarose gel prepared in 1× TBE (Tris-Borate-EDTA) buffer and stained with ethidium bromide at a concentration of 0.5 mg/mL. Electrophoresis was carried out at 90 V for 45 minutes. DNA bands were visualized under ultraviolet (UV) illumination, and fragment sizes were estimated by comparing the banding patterns to expected sizes based on the primer specifications.

### Designing specific primers of *S. oryzae*

The sequencing of the amplified PCR products was conducted to confirm the identity of the target insect pest species by analyzing the nucleotide composition of the *cytochrome oxidase* (*COX*) gene region. This process was carried out through a commercial sequencing service provided by Eurofins Genomics India Pvt. Ltd. The resulting amplicons were subjected to Sanger sequencing to generate precise DNA sequences of the targeted *COX* region. To verify the identity of the amplified sequences and ensure species-specific amplification, the obtained sequences were analyzed using the Basic Local Alignment Search Tool (BLAST) available on the National Center for Biotechnology Information (NCBI) website (https://www.ncbi.nlm.nih.gov/tools/primer-blast), using default parameters. The sequences were compared with publicly available *COX* gene sequences in the NCBI database to confirm their alignment with the cytochrome oxidase gene of *S. oryzae* and to check for any homology with unrelated species. This comparative analysis helped validate the specificity and accuracy of the amplified region. Species-specific primers for *S. oryzae* were designed by aligning *COX* gene sequences from *S. oryzae* and other insect species to identify conserved and unique regions. The alignment was performed using MEGA 11 (Molecular Evolutionary Genetics Analysis) software. The primary objective of this alignment was to identify conserved motifs within the *COX* gene suitable for designing primers capable of selectively amplifying *S. oryzae* DNA without cross-reactivity with non-target species. This strategy facilitated the development of new primers from conserved regions that allowed for precise and extended amplification of the *COX* gene, which in turn enabled reliable species-level identification following sequencing.

The newly designed forward and reverse primers were synthesized and procured from Eurofins Genomics India Pvt. Ltd. These primers were then used in subsequent PCR assays for species-specific amplification and molecular identification of *S. oryzae* in wheat grain samples.

### Amplification through polymerase chain reaction

The PCR amplification was carried out in a total reaction volume of 25 µL, prepared by combining 1 µL of template DNA (at a concentration of 50 ng/µL), 2 µL of custom-designed primer mix (comprising equal volumes of forward and reverse primers, each at 10 µM concentration), 12.5 µL of GoTaq^®^ G2 Green Master Mix (Promega), and 9.5 µL of nuclease-free water (NFW). A no-template control (NTC), containing all reaction components except the DNA template, was included to detect any contamination or nonspecific amplification. PCR amplification was performed using a Q Cycler 96 thermal cycler (Hain Life Sciences, UK). The thermocycling conditions included an initial denaturation at 95°C for 5 minutes, followed by 35 amplification cycles consisting of denaturation at 94°C for 30 seconds, primer annealing at 56°C for 30 seconds, and extension at 72°C for 1 minute. The final extension was carried out at 72°C for 10 minutes, after which the reaction mixtures were held at 4°C until further analysis. To confirm the amplification of the target cytochrome oxidase (*COX*) gene region, the PCR products were subjected to agarose gel electrophoresis. A 2% agarose gel was prepared in 1× TBE (Tris-Borate-EDTA) buffer and stained with ethidium bromide at a final concentration of 0.5 mg/mL. The gel was run at a constant voltage of 90 V for 45 minutes. The resulting DNA bands were visualized under ultraviolet (UV) light using a gel documentation system. Band sizes were estimated by comparing the migration pattern of the PCR amplicons to a standard 100 bp DNA ladder (Bangalore Genie, India), which served as the molecular weight reference. The presence of specific bands at expected sizes confirmed successful amplification of the target *COX* gene fragment.

### Specificity analysis

To verify the specificity of the designed primers, genomic DNA was also extracted from eight distinct unrelated species, including *R. dominica; Tribolium castaneum; Tribolium confusum*; *Callosobruchus chinensis*; *Oryzaephilus surinamensis*; *Lasioderma serricorne*; *Corcyra cephalonica*; and aphid*, Raphalosiphum maidis* using the previously described method.

Specificity of the designed primers towards *S. oryzae* was confirmed in standard PCR reaction as mentioned above by amplifying the designed primers using pure DNA of *S. oryzae* adults collected in eight different lots from the local market, contaminated and uncontaminated wheat grains and eight different unrelated insect species. NTC was kept for the experiment to check the specificity of the reaction. The PCR reaction cocktail, master mixture, thermal cycler profile, and electrophoretic conditions were similar as described earlier. This assay was replicated twice for confirmation.

### Sensitivity analysis

The sensitivity of the primers was evaluated using real-time PCR (qPCR). To detect *S. oryzae* contamination, DNA samples were extracted from both contaminated and uncontaminated wheat grains. This approach enabled quantitative assessment of *S. oryzae* infestation. Serial dilutions of the DNA were prepared, ranging from 10 ng/μl to 0.1 pg/μl, to assess the sensitivity of the primers. These DNA dilutions were used as templates for the qPCR experiments. Additionally, a NTC and DNA from healthy, uncontaminated grains were included to ensure specificity of the reactions. Pure *S. oryzae* DNA served as the positive control, while the NTC acted as the negative control. The DNA-binding fluorescent dye SYBR Green, in combination with the primers, provided high detection specificity during qPCR.

The qPCR reaction was carried out in a total volume of 20 µl, containing 1 µl of template DNA, 2 µl of the designed primer mix (equal volumes of forward and reverse primers), 10 µl of SYBR Green dye (Promega GoTaq^®^ G2 Green), and 7 µl of nuclease-free water (NFW). The reaction was conducted without DNA template for the NTC. The amplification protocol followed the same parameters as the conventional PCR experiments, and the reactions were performed using a Q Cycler 96 thermal cycler (Hain Life Sciences, UK). Cycle threshold (Ct) values were obtained for each DNA dilution. These results were validated by performing a melting curve analysis and constructing a standard curve. The efficiency of the qPCR was calculated by plotting Ct values against the logarithmic scale of DNA concentrations (in g) and determining the regression equation from the resulting graph.

## Results

### DNA quantity and quality

Concerning the amount and quality of DNA analysis using the BioDrop Touch PC + Spectrophotometer (BioDrop, Cambridge shire, UK), the majority of positive control DNA samples of test insect fell between 500 and 800 ng/μl, whereas the DNA extracted from grain samples fell between 800 and 2 μg/μl.

### Development of species-specific primers

Based on the multiple sequence alignment of *S. oryzae* accessions with sequences of other wheat-infesting insects available in the NCBI database, conducted using nucleotide BLAST and MEGA 11 software, two distinct primer sets were designed (**Table 2**). These two sets of forward and reverse primer pairs (KNSoCox1F1/KNSoCox1R1 and KNSoCox2F1/KNSoCox2R1) lie within the *COI* and *COII* region of *S. oryzae*, respectively.

**Table 2:** Primers developed in the present study specific to *COX I* and *COX II* region of *S. oryzae* DNA.

Primer Name		Forward and reverse primer sequences (5’-3’)	Primer base pairs	Amplicon size	T_a_ - Annealing temperature (˚C)
Primer 1	KNSoCox1F1	GAGCCCCAGATATAGCATTCC	21	176	56
	KNSoCox1R1	GGCCAGATCAACAGAAGCTC	20		
Primer 2	KNSoCox2F1	ATTGCCTTACCCTCACTTCG	20	248	56
	KNSoCox2R1	TCTGCAGACGTAACTAAGAGTCG	23		

### PCR detection and confirmation of diagnostic markers

In order to assess the KNSoCox1F1/KNSoCox1R1 and KNSoCox2F1/KNSoCox2R1 primer pairs efficacy, the genomic DNA extracted from eight distinct isolates of *S. oryzae* collected from the local market, the genomic DNA of contaminated and uncontaminated wheat grains, and the genomic DNA of eight distinct unrelated species (**Table 3**) were used as templates for the PCR assay. NTC was kept for the confirmation.

**Table 3:** Ct values of analyzed samples in real-time PCR reaction with primer 1 specific for *COI* of *S. oryzae* infesting wheat.

S.No.	Sample	Ct value
Primer 1		
1.	*S. oryzae*	18.83
2.	Contaminated wheat grains @ 1000 insects per kg	21.27
3.	Contaminated wheat grains @ 100 insects per kg	24.52
4.	Contaminated wheat grains @ 10 insects per kg	27.3
5.	Contaminated wheat grains @ 1 insects per kg	30.28
6.	Contaminated wheat grains @ 1 insects per 10 kg	33.12
7.	Uncontaminated grains	No Ct
8.	No DNA Template	No Ct
Primer 2		
1.	*S. oryzae*	17.92
2.	Contaminated wheat grains @ 1000 insects per kg	21.31
3.	Contaminated wheat grains @ 100 insects per kg	24.31
4.	Contaminated wheat grains @ 10 insects per kg	27.05
5.	Contaminated wheat grains @ 1 insects per kg	29.82
6.	Contaminated wheat grains @ 1 insects per 10 kg	33.21
7.	Uncontaminated grains	No Ct
8.	No DNA Template	No Ct

**Figure 1:**
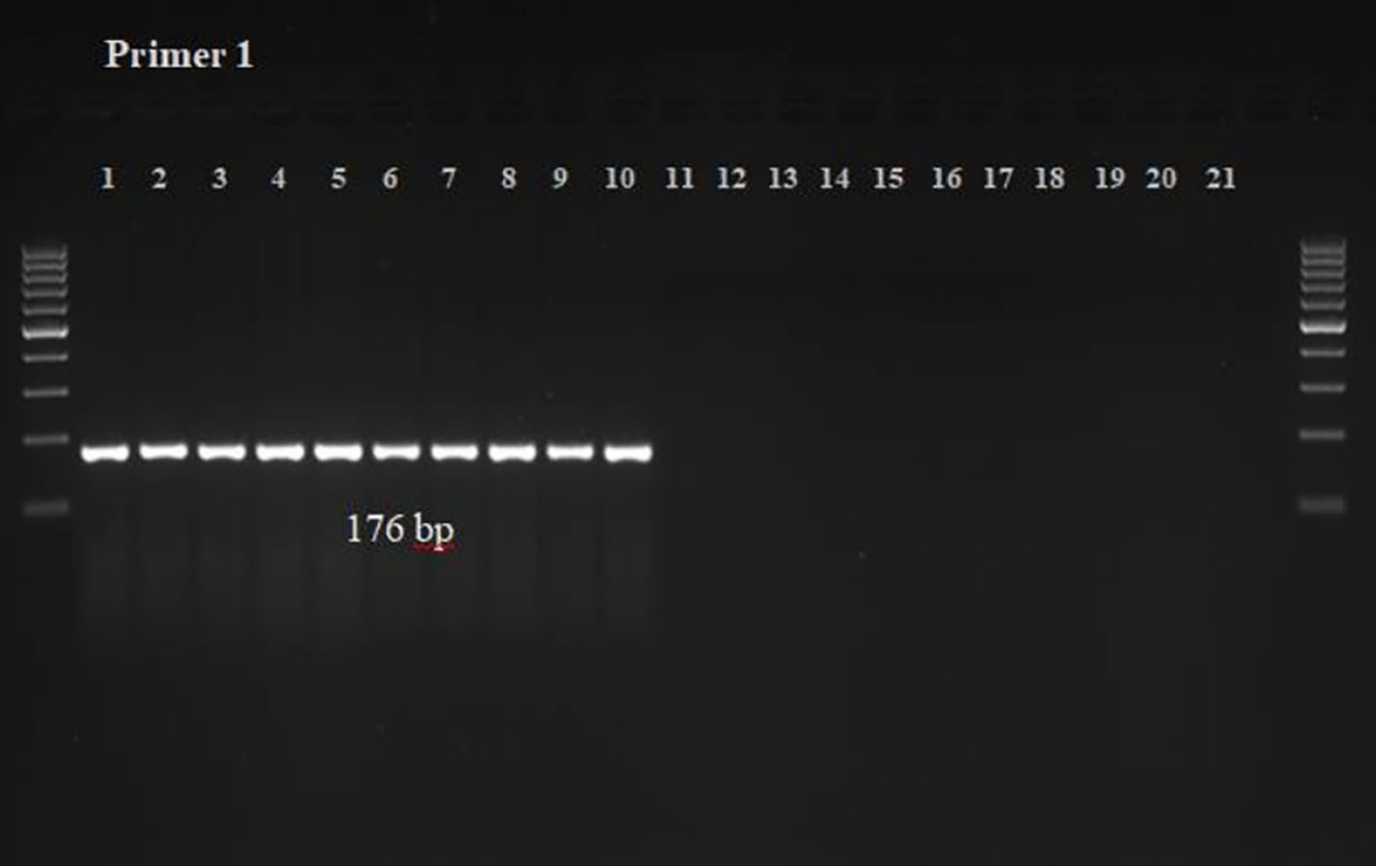
The PCR Reaction and specificity test for Primer 1 (KNSoCox1F1/KNSoCox1R1) specific to *COX I* region of *S. oryzae* DNA. The well numbering (1-21) in the figure represents the DNA samples of (1-8): *S. oryzae* obtained from eight different lots from local market; (9-10): *S. oryzae* infested/contaminated wheat grains; (11-12): uninfested healthy wheat grains; (13): *R. dominica*; (14): *Tribolium castaneum*; (15): *Tribolium confusum*; (16): *Callosobruchus chinensis*; (17): *Oryzaephilus surinamensis*; (18): *Lasioderma serricorne*; (19): *Corcyra cephalonica*; (20): aphid*, Raphalosiphum maidis*; (21): NTC.

**Figure 2:**
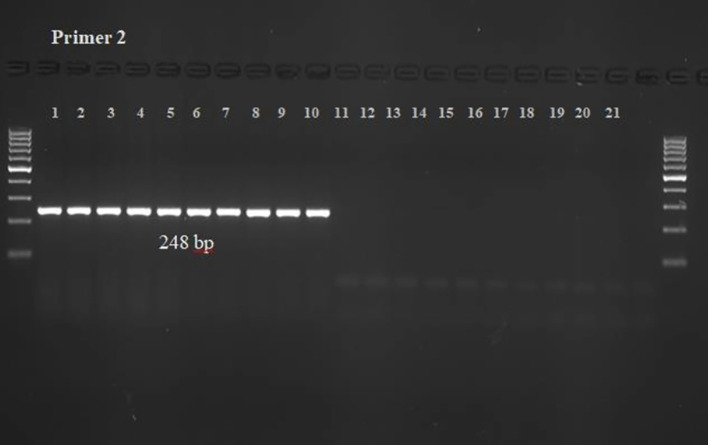
The PCR Reaction and specificity test for Primer 2 (KNSoCox2F1/KNSoCox2R1) specific to *COX II* region of *S. oryzae.* The well numbering (1-21) in the figure represents the DNA samples of (1-8): *S. oryzae* obtained from eight different lots from local market; (9-10): *S. oryzae* infested/contaminated wheat grains; (11-12): uninfested healthy wheat grains; (13): *R. dominica*; (14): *Tribolium castaneum*; (15): *Tribolium confusum*; (16): *Callosobruchus chinensis*; (17): *Oryzaephilus surinamensis*; (18): *Lasioderma serricorne*; (19): *Corcyra cephalonica*; (20): aphid*, Raphalosiphum maidis*; (21): NTC.

The chances of cross-species amplification for the developed PCR assay designed to detect *S. oryzae* infestation were negated by PCR-based amplicon generation (**Figures 1**, **2**). It showed that each set of the designed primers produced only a single band of 176 bp and 248 bp from the DNA of *S. oryzae* and contaminated grains, but not from the other 8 unrelated insect species, uncontaminated grains and NTC.

### Specificity and validation of diagnostic markers

The specificity of the designed primers for *S. oryzae* DNA and *S. oryzae*-infested wheat grains was assessed using a standard PCR reaction. *S. oryzae* DNA served as the positive control, and DNA with no template was used as the negative control. The results were verified through agarose gel electrophoresis, which displayed the amplified PCR products. The findings showed that primer 1 (KNSoCox1F1/KNSoCox1R1) targeting the *COX I* region and primer 2 (KNSoCox2F1/KNSoCox2R1) targeting the *COX II* region of *S. oryzae* DNA specifically bound at 176 bp and 248 bp, respectively, for both *S. oryzae* and contaminated wheat grains. No bands were observed for DNA extracted from uncontaminated grains or from eight other unrelated insect species (**Figures 1**, **2**). Consequently, these primers did not amplify DNA from insect-free grains or other insects. Additionally, the assay was repeated twice, and no significant variation in the results was found.

Therefore, the two primers that were found specific to *S. oryzae* were further tested through quantitative PCR (qPCR) to check the sensitivity of the primers by using different dilutions.

### Sensitivity analysis

**Figure 3:**
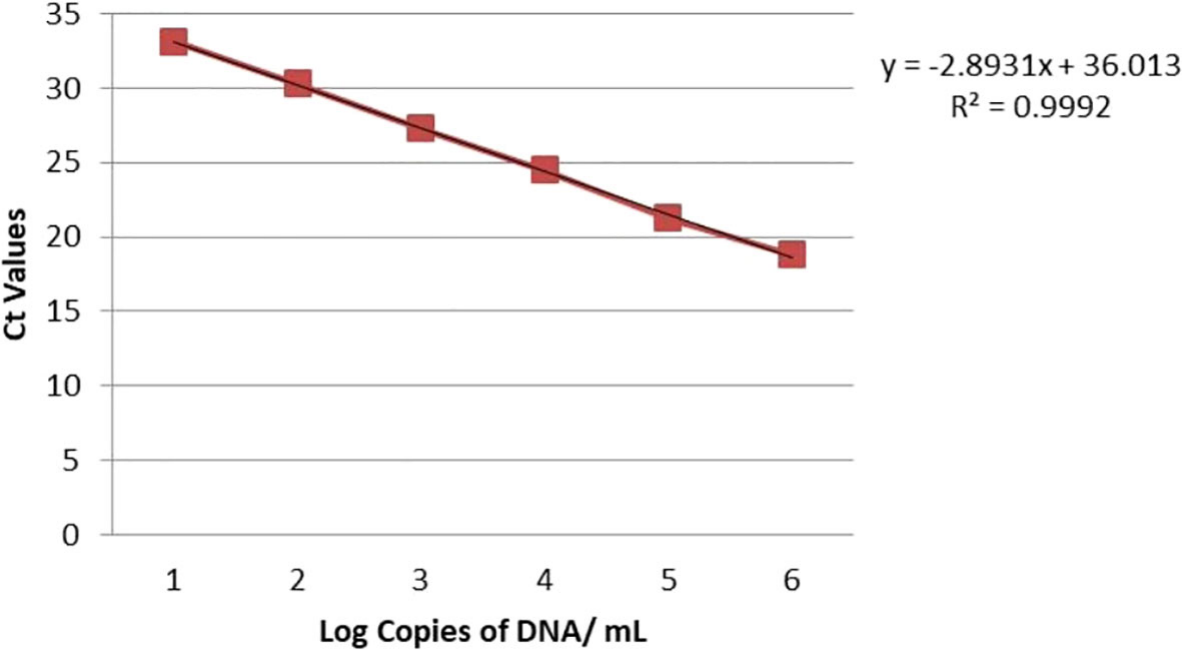
Standard curve of analyzed samples in real-time PCR reaction with primer 1 specific for *COI* of *S. oryzae* infesting wheat.

**Figure 4:**
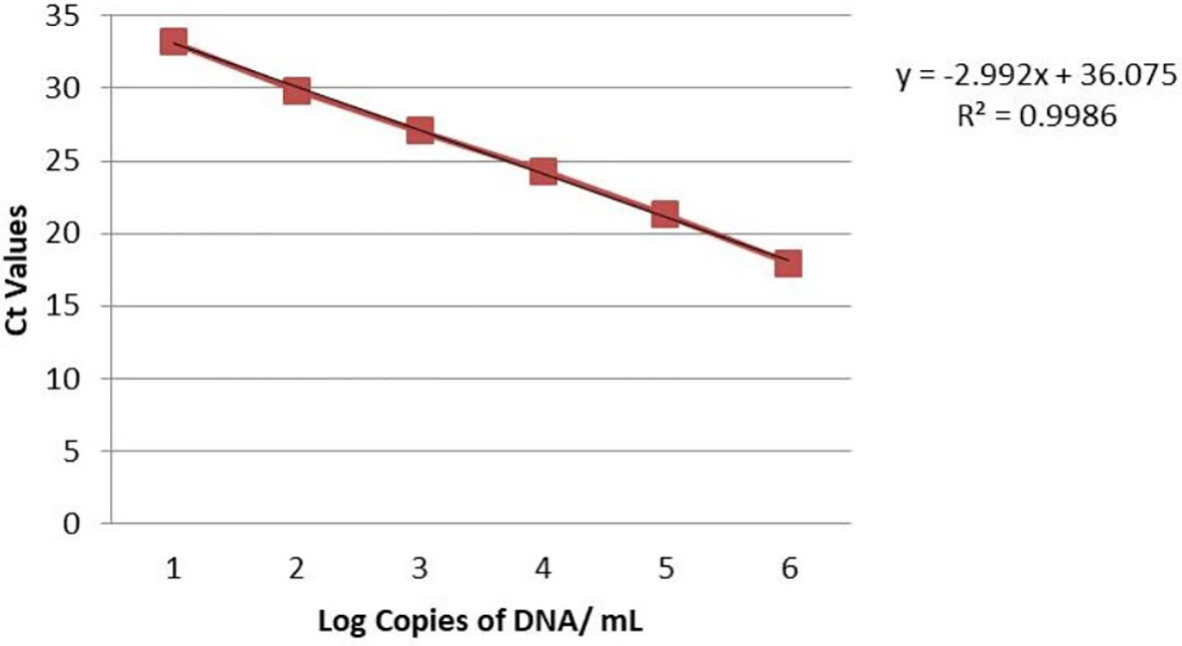
Standard curve of analyzed samples in real-time PCR reaction with primer 2 specific for *COII* of *S. oryzae* infesting wheat.

**Figure 5:**
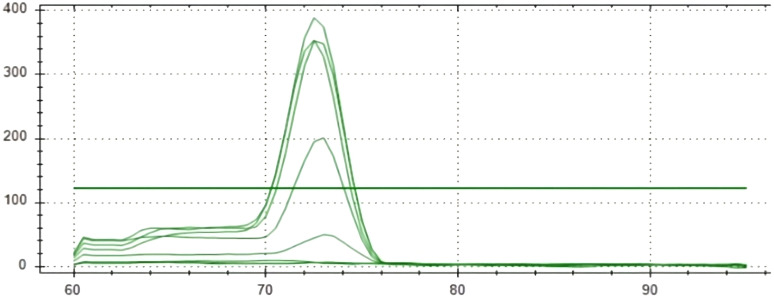
Melting curve of analyzed samples in real-time PCR reaction with primer 1 specific for *COI* of *S. oryzae* infesting wheat.

**Figure 6:**
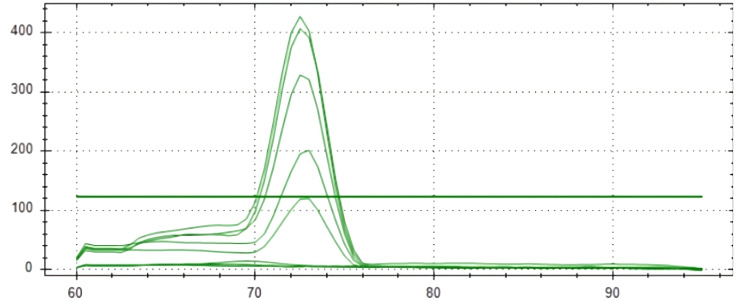
Melting curve of analyzed samples in real-time PCR reaction with primer 2 specific for *COII* of *S. oryzae* infesting wheat.

Real-time PCR was used to detect *S. oryzae* grain contamination using DNA samples isolated from contaminated and uncontaminated wheat grains. This approach makes it possible to assess the presence of *S. oryzae* infestation quantitatively. It makes it possible to even detect the *S. oryzae* DNA sample having infestation level of one insect per 10 kg of contaminated wheat grains for both the primers (Primer 1- KNSoCox1F1/KNSoCox1R1 and Primer 2- KNSoCox2F1/KNSoCox2R1). Ct (Cycle threshold) values were obtained for all these samples (Primer 1 & 2: **Table 3**). The Ct is defined as the cycle at which PCR enters the exponential phase and the fluorescence emission exceeds the threshold limit. The results were confirmed using standard curve (Primer 1: **Figure 3**; Primer 2: **Figure 4**) and melting curve (Primer 1: **Figure 5**; Primer 2: **Figure 6**) analysis. The efficiency of the qPCR was computed by graphing Ct values against -log (DNA concentration in g) and figuring out the regression equation.

## Discussion

Molecular biology-based diagnostic techniques are increasingly gaining traction across various biological disciplines, primarily due to their high reliability, sensitivity, rapid turnaround time, and specificity. In the present study, we developed and validated a rapid, sensitive, and species-specific molecular diagnostic method for the detection and identification of *S. oryzae* (rice weevil) infestation in wheat grains. *S. oryzae* is among the most destructive pests of stored cereals, particularly wheat, with considerable implications for post-harvest losses and international trade biosecurity.

To facilitate precise detection, we designed novel species-specific oligonucleotide primers targeting the *mitochondrial cytochrome oxidase* (*mtCOX*) gene complex, including both *COI* and *COII* subunits. These gene regions are widely recognized for their high interspecific variability and utility in molecular taxonomy and species-level identification. Although various genetic markers have been developed for the identification of grain-infesting beetles (Křížková-Kudlíková and Hubert, 2007), to our knowledge, this is the first report presenting *COI/COII*-based primers specifically designed for unambiguous detection of *S. oryzae* in wheat.

Early and accurate identification of *S. oryzae* infestations is critical for the enforcement of phytosanitary regulations, quarantine protocols, and surveillance measures during grain storage and international trade. PCR-based diagnostics have previously been developed for several storage pest species, including *Tribolium confusum* and *T. castaneum* (Nowaczyk et al., 2009; Kamel et al., 2016; Negi et al., 2021), as well as *S. zeamais* and *S. granarius* (Obrepalska-Steplowska et al., 2008; Devi et al., 2017; Solà et al., 2018). Moreover, a similar approach was imployed for the development of a highly sensitive (2.6 mg arthropod/500g rice) qPCR-based method for the detection of arthropod pests in stored rice by del Arco et al. (2025) using universal arthropod primers which successfully detected 10 most common pest species affecting rice but is recommended to be used only in commodities with a certain degree of processing to avoid environmental DNA detection. These PCR protocols are widely adopted in entomological diagnostic laboratories owing to their high sensitivity and specificity, minimal DNA requirements, and operational simplicity. Furthermore, commercial kits for DNA extraction from both insect tissue and grain matrices enhance the efficiency of molecular workflows. However, the reliability of this protocol is highly dependent on the effective strategies imployed for sampling and screening of the grain lots. For this method to detect the infestation, it is very important to ensure that multiple uniform sub-samples are taken, which truely represent the whole stock.

In this study, primer design was informed by computational analysis of the *mtCOX* gene region of *S. oryzae*. This locus is highly polymorphic among closely related taxa, making it an ideal target for species discrimination. Multiple studies have validated the effectiveness of *COX* genes for insect species identification (Ahrens et al., 2007; Nowaczyk et al., 2009; Kamel et al., 2016; Solà et al., 2018; Abbasi et al., 2021; Negi et al., 2021). The primers developed herein specifically amplify 176 bp and 248 bp fragments of the *COI* and *COII* regions, respectively. No amplification was observed from non-target insects or uninfested grains, affirming the assay’s high specificity. This is consistent with prior reports that underscore the importance of using highly variable mtDNA regions for reliable species discrimination (Min and Hickey, 2007; Dasmahapatra et al., 2010; Virgilio et al., 2012; Church et al., 2019).

The utility of the *mtCOX* region has also been extensively demonstrated in detecting pests such as *Plodia interpunctella*, *Rhyzopertha dominica*, *Oryzaephilus* spp., and *Samea* spp (Abbasi et al., 2021), as well as *T. confusum* (Nowaczyk et al., 2009) and *T. castaneum* (Negi et al., 2021). The use of multi-copy mitochondrial genes and variable nuclear ribosomal DNA regions (e.g., internal transcribed spacers or ITS) significantly enhances assay sensitivity (Cheng et al., 2003; Hsu et al., 2003). However, only regions exhibiting sufficient interspecific divergence are suitable for species-level resolution, as demonstrated by Peng et al. (2003) for closely related *Sitophilus* species. Kaundal et al. (2023) also used the *COX1* gene for molecular analysis to validate the presence of *S. oryzae* and *S. granarius*. Similarly, the existence of *S. oryzae*, *S. zeamais* and *S. granarius* was confirmed by Suhriani et al. (2023) using *COX1* gene.

Our PCR-based approach offers notable advantages over conventional detection methods. It requires only standard laboratory equipment—thermal cyclers and electrophoresis systems—making it feasible for routine implementation. The assay demonstrated exceptional sensitivity, detecting as little as 1 picogram (pg) of *S. oryzae* genomic DNA, equivalent to the infestation of a single insect per 10 kg of wheat grain, aligning with detection thresholds reported by Nowaczyk et al. (2009). This is particularly significant in the context of the Polish standard PN-69/R-74016, which allows only one insect per kilogram of grain.

Moreover, a quantitative real-time PCR (qPCR) assay was also developed to assess infestation levels. This approach enhances diagnostic resolution, enabling detection of minute quantities of target DNA, even from residual insect fragments in sieved grain samples. The qPCR protocol, using *COI/COII*-specific primers, allows for the establishment of standard curves and accurate quantification of *S. oryzae* DNA across a range of concentrations. This provides a robust supplementary tool for surveillance and contamination assessment in grain storage facilities.

The developed PCR assay is of high practical relevance for quarantine and regulatory applications where time-sensitive and precise species identification is imperative. Notably, it permits rapid screening of samples within 24 hours, enabling timely decision-making and mitigation strategies. Importantly, the assay is capable of detecting *S. oryzae* infestation at an early stage, thus facilitating prompt intervention and reducing post-harvest losses.

## Conclusion

In this study, two species-specific molecular markers targeting the mitochondrial *COI* and *COII* genes of *S. oryzae* were developed using primer pairs KNSoCox1F1/KNSoCox1R1 and KNSoCox2F1/KNSoCox2R1. Standard PCR assays produced distinct amplicons of 176 bp and 248 bp, confirming high specificity with no cross-reactivity in non-target insects or uninfested grains. Real-time PCR detected *S. oryzae* DNA at infestation levels as low as one weevil per 10 kg of wheat. The assay is rapid, sensitive, reliable, and cost-effective, making it ideal for entomological diagnostics, ecological studies, host-pest interactions, routine screening of stored products, and quarantine applications. It provides a valuable tool for early detection and quantification of *S. oryzae* in pest management and biosecurity.

## Data Availability

The datasets presented in this study can be found in online repositories. The names of the repository/repositories and accession number(s) can be found in the article/supplementary material.
